# Anticholinergic burden and comorbidities in patients attending treatment with trospium chloride for overactive bladder in a real-life setting: results of a prospective non-interventional study

**DOI:** 10.1186/s12894-018-0394-8

**Published:** 2018-09-14

**Authors:** A. Ivchenko, R.-H. Bödeker, C. Neumeister, A. Wiedemann

**Affiliations:** 1 Department of Urology, Evangelisches KrankenhausWitten gGmbH, UniversityWitten/Herdecke, Pferdebachstrasse 27, 58455 Witten, Germany; 2 Department of Statistics, Institute of Medical Informatics, University Clinic Giessen, Rudolf-Buchheim-Strasse 6, 35392 Gießen, Germany; 3 Department of Medical Science/Clinical Research, Dr. R. Pfleger GmbH, Dr.-Robert-Pfleger-Strasse 12, 96052 Bamberg, Germany

**Keywords:** Anticholinergic burden, Comorbidity index, CIRS-G, Overactive bladder, Elderly patients, Trospium chloride, Non-interventional study

## Abstract

**Background:**

Elderly people are representative for the patients most likely to be treated with anticholinergics for overactive bladder (OAB). They often receive further drugs with anticholinergic properties for concomitant conditions. This increases the risk for side effects, including central nervous system disorders. Data on comorbidities and baseline anticholinergic burden of OAB patients seen in urological practice is scarce. Therefore, we included an epidemiological survey on these issues in our study which assessed the effectiveness and tolerability of trospium chloride (TC) in established dosages under routine conditions.

**Methods:**

Outpatients (≥ 65 years of age), for whom treatment with TC was indicated, were eligible to participate in this non-interventional, prospective study performed in 162 urological practices in Germany. Epidemiological questions were evaluated by the Anticholinergic Burden (ACB) scale and the Cumulative Illness Rating Scale for Geriatrics (CIRS-G) at baseline. Efficacy was assessed by changes in symptom-related variables of OAB after treatment. Dosage regimen, duration of treatment, adverse events, withdrawals, and ease of subdivision of the prescribed SNAP-TAB tablet were documented. Patients and physicians rated efficacy and tolerability of treatment. Statistics were descriptive.

**Results:**

Four hundred fourty-five out of 986 (47.54%) patients in the epidemiological population had a baseline ACB scale score > 0, 100 (24.72%) of whom a score ≥ 3. The median CIRS-G comorbidity index score for all patients was 5. 78.55% (608/774) of patients in the efficacy population received a daily dose of 45 mg TC. 60.03% (365/608) of them took this dose by dividing the SNAP-TAB tablet in three equal parts. Before-after-comparisons of the core symptoms of OAB showed clear improvements. An influence of the dosage scheme (1 × 45 mg TC/d vs 3 × 15 mg TC/d) on clinical outcome could not be observed. Most urologists and patients rated TC treatment as effective and well tolerated. 44 (4.37%) out of 1007 patients in the safety collective ended their treatment prematurely, while 75 patients (7.45%) experienced adverse events.

**Conclusions:**

Anticholinergic burden and comorbidities in elderly OAB patients are frequent. The acceptance of the SNAP-TAB tablet, which facilitates flexible dosing with TC, was high, which is supportive in ensuring adherence in therapy.

**Trial registration:**

This non-interventional study was registered on October 29, 2014 with the number DRKS00007109 at the German Register of Clinical Studies (DRKS).

## Background

Trospium chloride, a synthetic quaternary antimuscarinic, is intended for symptomatic treatment of the overactive bladder syndrome, providing patients with a fast, reliable and considerable improvement or cure of the stressful symptoms: urinary incontinence, urgency and frequency [1–6]. The recommended daily dose is 45 mg TC (3 × 15 mg).^1^ It is adequate for most patients with OAB. Flexible dosing up to daily doses of 90 mg TC is safe and well tolerated, permitting treatment to be tailored to the patient’s optimal individual balance between efficacy and side effects [1, 7–9].

Increasing attention is paid to safety and compliance concerns, as older people show an increased prevalence of gradually declining human organ and body functions, resulting in physical, physiological and/or cognitive impairments, multi- and co-morbidities, and/or frailty [10]. They are exposed to an increasing number of medications (polypharmacy), often with known or unknown anticholinergic activity, including prescriptions and over-the-counter products [10, 11]. Estimates suggest that one third to half of commonly prescribed drugs for the elderly have anticholinergic properties [12]. Due to the pattern of receptor distribution and their mechanisms of action, anticholinergic drugs as well as many other drugs not usually denoted as anticholinergics, show their anticholinergic activity throughout the human body. This is often associated with a variety of adverse effects (AEs). The most common are peripheral AEs, such as dry mouth, blurred vision, constipation, and tachycardia, as well as central nervous system (CNS) AEs, including dizziness, sedation, falls, confusion, delirium and cognitive impairment [12–20]. These can, in turn, further worsen patient’s mental and physical health status, often leading to dependence [21]. On the other hand, OAB is a common yet disabling condition with a considerably negative impact on the patient’s quality of life, sleep, sexual function, work productivity and general mental health [11, 22–24]. Therefore, it is often associated with a significant increase in troublesome symptoms and comorbidities in those patients, such as falls, urinary tract infections, hypertension, diabetes [25–27], as well as higher odds for loneliness and depression [26, 28–30]. The causal relationship between many of these comorbid conditions as well as the question whether treatment of one condition improves or exacerbates the other have thus far largely remained unclear [26]. Nevertheless, muscarinic receptor antagonists are universally accepted to be the first-line pharmacotherapy of OAB [31, 32].

Physicians prescribe drugs with primary or secondary anticholinergic properties based on their anticipated therapeutic benefits. Herein, they sometimes overlook that the concurrent use of several drugs with anticholinergic properties likely results in cumulating effects in the vulnerable elderly patients [12, 15, 21, 33]. This so-called anticholinergic burden (or anticholinergic load) can adversely impact both cognitive and functional status of patients further. Moreover, elderly individuals are thought to be particularly vulnerable to central nervous system AEs of anticholinergics due to age-associated morphological, biochemical, physiological and pathological changes in the brain [12, 34, 35]. A comprehensive systematic review examining associations between drugs with anticholinergic properties and adverse outcomes in older adults concluded that exposure to certain individual drugs with anticholinergic effects or increased overall anticholinergic exposure may increase the risk of falls, cognitive impairment and all-cause-mortality in these patients [36]. Therefore, physicians should carefully consider medical history and concomitant medications when initiating antimuscarinic treatment of OAB in elderly patients.

Generally, however, on the comorbidities and the baseline anticholinergic burden of OAB patients seen in daily urological practice very little information exists. To address this gap of knowledge, we included an epidemiological survey on these two issues in a non-interventional study (NIS) assessing treatment responses and tolerability to TC administered according to current routine treatment schemes in a diverse population of OAB outpatients. Evaluating the ease of subdivision of the SNAP-TAB tablet preparation, containing 45 mg of TC, by the elderly participants was a further objective of this study.

## Methods

### Study design

This open, prospective, observational study was conducted in 162 urology practices in Germany between November 2014 and October 2015. The number of patients that could be recruited by a single centre was limited to 10 to ensure that the data was not predominantly generated by few large practices, which could jeopardize the representativeness of the sample. The selection and number of study centres was set as is to reflect the most representative picture of “medical practice” in Germany possible. Regarding the data to be gathered in the epidemiological part of the study, 1250 patients were to be recruited into this study to obtain a final sample size of approximately 1000 patients for the analysis of epidemiological research questions. The TC therapy was prescribed by the participating urologists in the course of normal outpatient care, was commercially available and funded according to local practice in usual routine care. The study protocol, therefore, did not contain any specifications regarding dosing of TC or duration of treatment. Instead, the advising urologists were asked to follow the recommendations defined in the licensed approval by the national regulatory authorities. The contraindications, special warnings, precautions for use, interactions, information on use during pregnancy and lactation, effects on ability to drive and use machines, as well as undesirable effects specified in the Summary of Product Characteristics (SmPC) had to be observed.

### Compliance with ethics

This post-registration trial conforms with § 67(6) of the German Drug Law. All procedures were carried out in accordance with the official recommendations regarding the conduct of non-interventional studies by the Federal Institute for Drugs and Medical Devices (BfArM) and the Paul-Ehrlich-Institute [37], and the recommendations for assuring Good Epidemiological Practice [38]. Accordingly, the study was notified to the federal authority and the relevant associations. Approval of an ethical committee was not required for such a non-interventional trial in Germany [§67(6) of the German Drug Law]. Nevertheless, the study protocol was submitted to the Ethics Committee of the Medical Chamber Westphalia-Lippe and the Westphalian Wilhelm University Münster, Germany, which gave a favourable recommendation prior to the start of the study (September 2014). All data and information collected in the scope of this study was gathered in accordance with the recommendations for baseline diagnosis specified in the AWMF Guideline No. 084/001 *Urinary Incontinence,* published by the German Geriatric Society [39]. The study was performed within the indication approved in the marketing authorization and under consideration of the contraindications and precautions defined therein [40]. Each physician had to decide on the OAB-therapy independently from the assignment of a patient’s inclusion into the study. Patients were admitted only after they had given their written consent to the data protection policy at the first visit. Participants were free to withdraw at will at any time without giving reasons and without incurring disadvantages. Documentation of study-related data of each patient was performed solely in accordance with routine urological assessments.

### Patients and treatment

Elderly men and women (≥ 65 years of age) with symptoms of OAB for whom the attending urologist had decided to prescribe a TC preparation containing 45 mg active agent per tablet (Spasmex® 45 mg film-coated tablets) were included in this trial. Consistent with the non-interventional nature of the study, no further restrictions were applied in respect to the inclusion of patients or to the dose and duration of treatment. The preparation Spasmex® 45 mg is a modern SNAP-TAB tablet designed for easy and precise subdivision [41, 42].

### Assessments

Participation in this trial included three visits per patient, defined as first, interim and last visit (= Visit 1, 2, 3), with a recommended minimum interval between visits of 10 days. The minimum duration of treatment was recommended to be no less than 6 weeks.

At Visit 1, patients were questioned regarding their comorbidities and their anticholinergic burden, using established scales and questionnaires. The Anticholinergic Cognitive Burden Scale, adapted to the German market, was selected to measure anticholinergic burden [19, 43, 44]. The level of burden caused by chronic illnesses was assessed using the German version of the Cumulative Illness Rating Scale for Geriatrics, which rates the severity of chronic diseases in 14 organ-specific categories on a five-point scale of 0 to 4 [45]. The illness ratings across all organ categories are subsequently summed up to create the comorbidity index (CMI).

The attending urologists entered the patient’s data online via an encrypted website, using a validated German Internet-based input system (portal of MedSurv GmbH, Nidderau, Germany). They collected the following information at the specified time points and/or, if applicable, at the time of premature discontinuation of treatment: demography, medical history, pre-treatment of OAB, anticholinergic burden-related medications contributing to the ACB scale score, concomitant diseases contributing to the baseline CIRS-G score, further relevant comedication, OAB symptoms (number of voids/24 h, number of nocturnal voids/hour sleeping time, severity of urgency symptoms, occurrence and number of incontinence episodes, usual amount of urine leakage), prescribed dose and timing of administration of TC 45 mg, any adverse events as well as premature treatment termination. At Visits 2 and 3, physicians and patients were asked to assess effectiveness and tolerability of therapy using the following four categories: very good, good, poor, very poor. Additionally, at Visit 3 or time of premature discontinuation of treatment the investigator queried the patient to rate the ease of subdivision of the SNAP-TAB tablets on a four-point scale with the categories: very easy, easy, somewhat difficult, very difficult.

### Data management and statistics

The validity of the anonymized submission data was checked for plausibility and completeness. Missing data on patient and physician assessments of treatment efficacy and tolerability and variables describing OAB symptoms at the last visit was replaced by the corresponding data collected at the interim visit in cases where the time difference between the first and the interim visit was at least 10 days. Adverse events were classified using the MedDRA coding system V19.1.

Transformation, preparation and exploratory analyses of data were carried out using the statistical software package SAS® V9.3 and 9.4, respectively. Since direct calculation of the exact confidence interval of the median was not possible with SAS, it was done using the DescTools package in R-version 3.3.1. The IML module was used to call R from SAS.

Data was analysed using descriptive statistical methods. The distributions of the qualitative and discrete quantitative variables were described in terms of absolute and relative frequencies based on sample size of the respective collective and were presented by three age classes and gender separately, and globally for all patients. The distributions of the continuous variables and quantitative discrete variables with a lot of values were described by sample size, number of missing values, minimum, 1. quartile (Q1), median, 3. quartile (Q3), maximum, and confidence interval (CI) of the median.

To answer the epidemiological research questions, we used logistic regression methods. Since the target variable, the ACB scale score, was extremely skewed to the right, we divided the variable “ACB score” into two classes (ACB = 0 and ACB > 0) before evaluating the data. Since a linear influence of age and CIRS-G score-derived comorbidity index on the probability of the presence of an anticholinergic burden could also not be assumed, the explanatory variable “age” was divided into three classes (65 years to < 75 years, 75 years to < 85 years, and ≥ 85 years), and the explanatory variable “comorbidity index” was divided into four quartile classes (0–2, 3–4, 5–7, and ≥ 8). Effects of the potential effect modifiers age, gender and CIRS-G derived comorbidity index as a measure of the number of health problems were then studied using logistic regression. Because the comorbidity index was also extremely right-skewed, we also transformed this index into a dichotomous variable (≤ 4 versus > 4) when handling the age−/gender-related question to the presence of comorbidity burden.

Efficacy outcomes were evaluated by change in the quantitative variables describing OAB symptoms which was defined as the score difference between the respective variable at Visit 1 and by using a new target variable constructed using the combined data from Visits 3 and 2 (last evaluable Visit minus Visit 1).

### Post hoc subgroup analysis

**Fig. 1:**
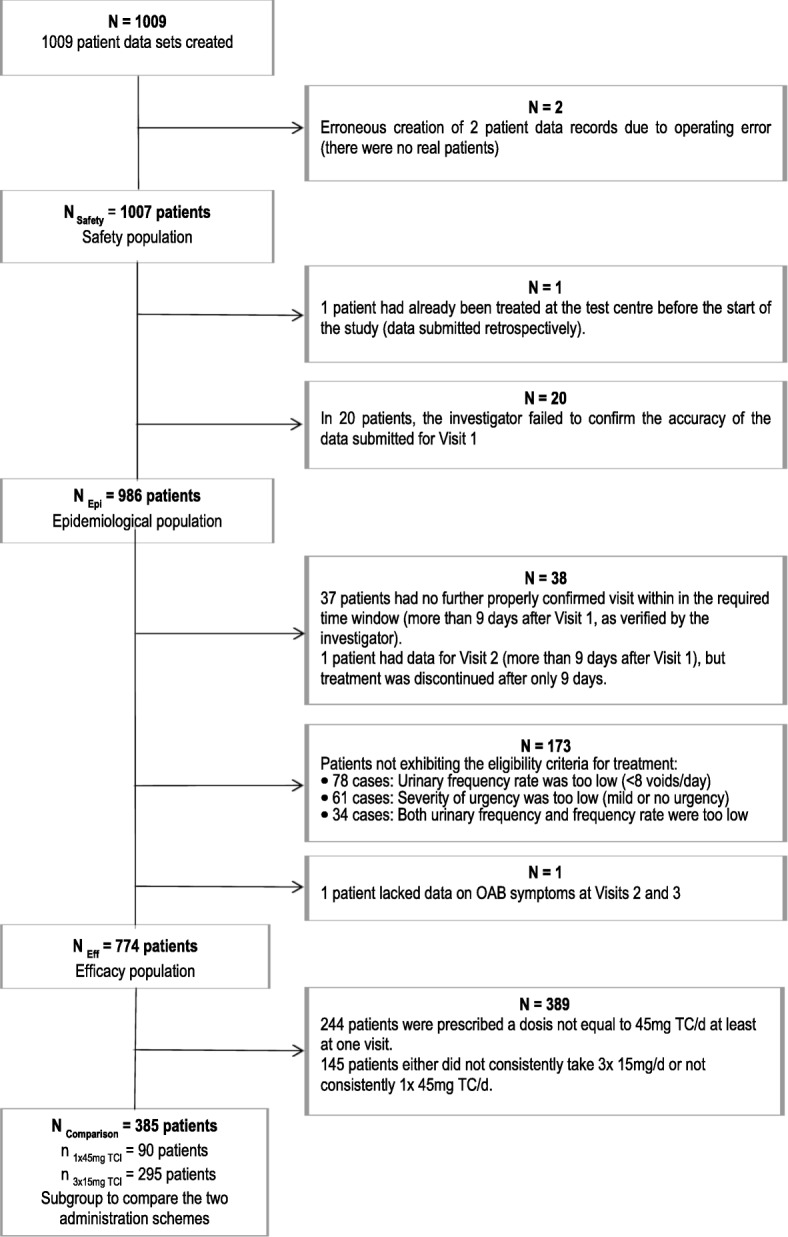
Assignment of patients to the analytical populations

After the statistical analysis was finished as laid down in the study protocol, a subgroup of the efficacy analysis set (*n* = 385 patients) consisting of two treatment groups according to the dosage regime “1 times a day 45 mg of TC” (*n* = 90 patients) or “3 times a day 15 mg of TC” (*n* = 295 patients) documented over the whole treatment period, was used to compare treatment outcomes in relation to the two administration schemes (Fig. 1). All analyses were descriptive and explorative in nature.

For between-group comparisons relating the main variables, we used effect size measures for evaluating the strength of the observed result. We calculated *Cohen’s r* for the median *change of average number of voids/24 h between Visit 1 and the last evaluable visit*; and *Cramér’s V* for the *combination in the occurrence of incontinence episodes at Visit 1 to the last evaluable visit*, as well as for the assessments of efficacy and tolerability by physicians and patients. For facilitating the interpretation of effect sizes, we used the defined reference values by Cohen [46] and Ellis [47].

## Results

### Participants

All 1007 recruited participants, including one patient who had been treated before the start of the study and whose data had been submitted retroactively, were included in the safety analysis. In accordance with the study protocol, data sets from 986 patients were available for the epidemiological analysis, and 774 were available for the efficacy analysis (Fig. 1).

### Epidemiological population and research-related characteristics

Out of the 986 patients, 564 (57.2%) were women and 422 (42.8%) were men (Table 1). The median age in this population was 75.0 years (range: 65–97; Q1: 70.0, Q3: 79.0) with equal distributions by gender (Hodges-Lehmann estimator: 1 year, 95% CI: [0 years, 2 years]).

**Table 1:** Epidemiological population: Frequency distribution of age classes at the first visit, by sex, and globally

Sex	Patient age at Visit 1 (3 classes)						Total
	65 years to < 75 years		75 years to < 85 years		≥ 85 years		
	n	%	n	%	n	%	N
Female	281	49.82	239	42.38	44	7.80	564
Male	184	43.60	192	45.50	46	10.90	422
All patients	465	47.16	431	43.71	90	9.13	986

**Fig. 2:**
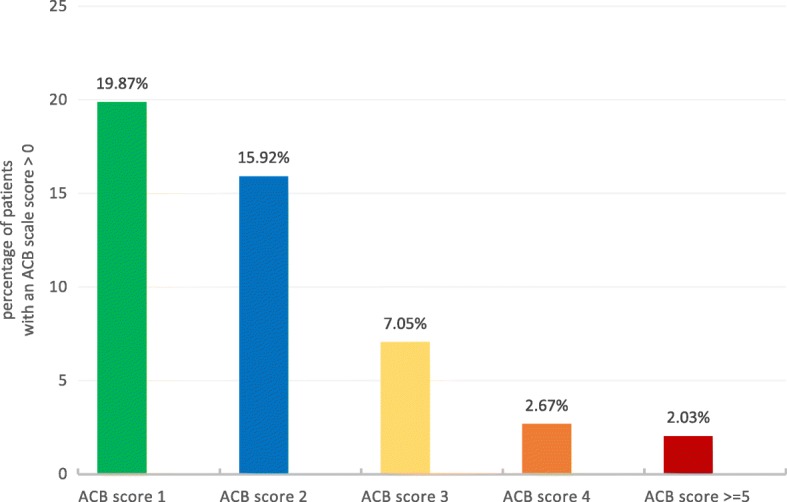
Frequency distribution of patients with an ACB scale score > 0 at baseline (*n* = 445). The percent data refer to 936 patients of the epidemiological population. An ACB scale score of ≥3 was considered clinically relevant [19]

At Visit 1, data of 936 patients was analysed and scored on the ACB scale. Overall, 491 (52.46%) patients in the epidemiological population were not taking any drugs with potential anticholinergic effects, as reflected by an ACB scale score of 0; while 445 patients (47.54%; 95% CI [44.30%; 50.80%]) of all patients with evaluable ACB score data at Visit 1 had a baseline anticholinergic burden, as defined as an ACB scale score of > 0 (Fig. 2). The total number of ACB-related drugs taken by the participants was 657. Of them, 479 (72.91%) had an ACB score of 1, 115 (17.50%) had a score of 2, and 63 (9.59%) were anticholinergics with an ACB score of 3. The most commonly used anticholinergic medication was metoprolol (*n* = 159), followed by TC (*n* = 101) and furosemide (*n* = 66).

The median comorbidity index, as calculated from the CIRS-G data for all patients of the epidemiological population, was 5 (exact 95% CI [4.0%; 5.0%]; sum of items 1–14, observed range 0–33), with 52.03% (513/986) of patients observed with a CMI of > 4. Most patients (837/986, 84.89%) did not have any relevant somatic morbidity (RSM, number of items with a rating of 3 or 4, except for psychiatric disorders = 0), 11.16% of patients (110/986) had a RSM of 1, 3.25% (32/986) a RSM of 2, and 0.71% (7/986) a RSM score between 3 and, at maximum, 5.

The data analysis of possible effect modifiers for the ACB score indicated that the chances of having an anticholinergic burden (ACB > 0) might be higher in patients aged ≥85 years and seemed to be higher in men than in women; additionally, the odds of having an anticholinergic burden seemed to increase with an increasing CMI (Table 2). Similarly, regarding the CIRS-G score-related comorbidity index in this population, the occurrence of having a CMI of > 4 seemed to increase with increasing age and women might have a lower likelihood than men.

**Table 2:** Epidemiological population: Effects of age and sex on ACB score and CMI

Effect	Category of interest versus reference category	Point estimate of the odds ratio	95% confidence interval (Wald’s type)
A. Effects of age, gender and/or CIRS-G score-related comorbidity index on ACB score			
Sex	Female vs. male	0.72	[0.55; 0.94]
Age class	75 years to < 85 years vs. 65 years to < 75 years	1.13	[0.85; 1.50]
	≥ 85 years vs. 65 years to < 75 years	1.79	[1.08; 2.94]
CMI (4 classes)	Quartile 2 CMI 3–4 vs. Quartile 1 CMI 0–2	1.69	[1.13; 2.52]
	Quartile 3 CMI 5–7 vs. Quartile 1 CMI 0–2	2.03	[1.38; 2.99]
	Quartile 4 CMI ≥ 8 vs. Quartile 1 CMI 0–2	3.29	[2.23; 4.86]
B. Effects of age and/or gender on the comorbidity index			
Sex	Female vs. male	0.57	[0.44; 0.74]
Age class	75 years to < 85 years vs. 65 years to < 75 years	2.28	[1.74; 2.99]
	≥ 85 years vs. 65 years to < 75 years	2.79	[1.73; 4.51]

Odds ratio point estimates and confidence intervals calculated by comparing the respective category of interest with the reference category for the possible explanatory variables included in the model for the likelihood of having an anticholinergic burden (A) or a comorbidity index of > 4 (B)

### Evaluation of efficacy

The population of the efficacy analysis set comprised 774 patients, 456 women and 318 men, with a median age of 75.0 years (range: 65–97; Q1: 70.0, Q3: 79.0) and equal distributions by sex (Hodges-Lehmann estimator for the difference between the location: 1 year, 95% CI: [0 years, 2 years]).

At study entry, 256 out of 764 patients (33.51%) suffered from OAB symptoms for years, 408 patients (53.40%) for months, and 100 patients (13.09%) reported symptoms occurring during the last weeks or days. 34.37% (266/774) of patients had received medical pre-treatment for their OAB syndrome; most frequently used drugs were anticholinergics (62.41%) and herbal drugs for urological disorders (11.28%). The reasons for switching of medication were “insufficient efficacy” in 221 out of 267 cases (82.77%) and “lack of tolerance” in 31 cases (11.61%).

**Fig. 3:**
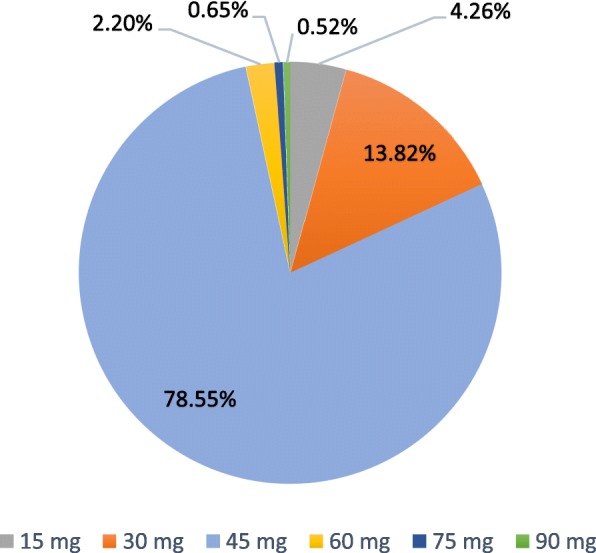
Prescribed daily doses of TC (mg/day) at Visit 1 – Efficacy population (*n* = 774)

At Visit 1, 78.55% (608/774) of patients were instructed to take a daily dose of 45 mg TC (Fig. 3). Of them, 60.03% (365/608) took the prescribed dose by dividing the SNAP-TAB tablet in three equal parts corresponding to 15 mg of TC each, to be taken in the morning, noon and night; 19.41% (118/608) took the whole 45 mg tablet as a single daily dose, and 20.56% (125/608) divided the tablet in two doses, one of 30 mg and one of 15 mg of TC.

**Fig. 4:**
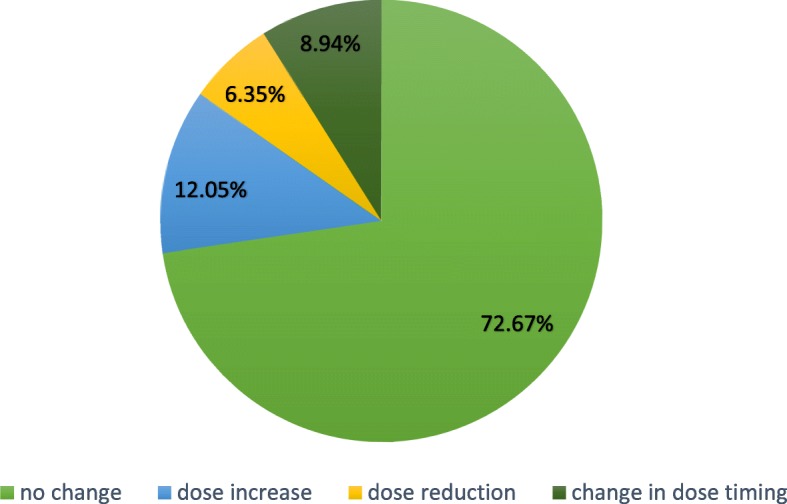
Changes in dose or dosage regimen at Visit 2 (*n* = 772/774) – Efficacy population

At Visit 2, the physician decided on the patient’s individual response to treatment whether a dose adjustment and/or a change in dosage regimen was necessary or not. In 81.61% (630/772) of patients, the prescribed daily dose remained unchanged (Fig. 4).

The median treatment period documented at the last evaluable visit (*n* = 774) was 64 days (min = 10 (predefined), max = 325; Q1: 46, Q3: 98). Treatment with TC improved symptoms of OAB as evaluated by before-and-after comparisons of different variables. The median change in the average *number of voids per 24 h*, as calculated by subtracting the outcome for Visit 1 from that for the last evaluable Visit, was − 4 (Q1: -6, Q3: -2; exact 95% CI [− 4 voids/24 h; − 4 voids/24 h]) for all patients included in the efficacy population (n = 774). The data analysis by logistic regression indicated that women were more likely to have a ≥ 4-void reduction in the average number of voids per 24 h than men (female vs. men: point estimate of the odds ratio (OR) 1.41, 95% CI_Wald’s type_ [1.05; 1.91]). Moreover, patients aged ≥75 years to < 85 years seemed to have a lower chance to experience this improvement than patients aged ≥65 years to < 75 years: OR 0.71 (95% CI_Wald’s type_ [0.52; 0.97]). The median change in the average *number of nocturnal voids/hour sleeping time in the last 7 days* was − 0.3 (Q1: -0.4, Q3: -0.1; *n* = 769). Under treatment, the *severity of urgency symptoms* decreased in 655 (84.63%, 95% CI [81.89%; 87.10%]) out of 774 patients.

38.50% (298/774) of the patients included in the efficacy analysis did not have any incontinence episodes by the time of the first and the last visit. Improvement in the *occurrence of incontinence episodes* was observed in 231 (29.84%, 95% CI [26.64%; 33.21%]) out of 774 individuals, 528 (68.22%) showed no change, and 15 (1.94%) reported worsening of accident occurrence. The *number of incontinence episodes* in this population (*n* = 470) decreased by a median of 5 incontinence episodes (Q1: -10, Q3: -2; 95% CI [− 5.0; − 4.0]). Six further patients confirmed the occurrence of incontinence episodes in the last 7 days before the respective visit but did not provide valid data describing the number of incontinence episodes or the amount of urine leakage. In 349 cases (74.26%) the *amount of urine leakage* decreased from Visit 1 to the last evaluable visit.

The post hoc subgroup analysis did not indicate a difference in treatment outcome between the two dosage regimens “1 x 45 mg TC/day” and “3 x 15 mg TC/day” for any of the efficacy variables. This was also obvious from the effect size measures relating to the variables, with *Cohen’s r* = 0.103 for the median *change in the number of voids/24 h between Visit 1 and the last evaluable visit,* and *Cramér’s V* = 0.050 for the *combination in the occurrence of incontinence episodes at Visit 1 to the last evaluable visit.* As the effect size of any variable was < 0.1 or only marginally greater than 0.1, this had to be considered a trivial effect in accordance with the conventions by Cohen [46] and Ellis [47].

### Global assessment of effectiveness and tolerability

At the last evaluable visit, 91.21% of the physicians (706/774) assessed the effectiveness of the OAB treatment with TC 45 mg tablet as either “very good” or “good”, as did 89.53% of patients (693/774). The question relating to therapy continuation with 45 mg of TC was answered affirmatively for 672 patients (89.36%); 57 patients (7.58%) required no further treatment and 23 (3.06%) were switched to another treatment modality.

Tolerability was assessed predominantly “very good” or “good” by 94.32% (730/774) of the physicians and by 90.96% (704/774) of the patients.

This trend was also observed in the post hoc analysis regarding the global assessment of effectiveness and tolerability across the two analysed dosage regimens; effectiveness: physicians – *Cramér’s V* = 0.122, patients – *Cramér’s V* = 0.161; tolerability: physicians – *Cramér’s V* = 0.075, patients – *Cramér’s V* = 0.107. Based on the reference values for effect size measures by Ellis [47], this indicated that the observed effect differences between the two treatment regimens were either irrelevant or very small at maximum.

### Ease of subdivision analysis

**Fig. 5:**
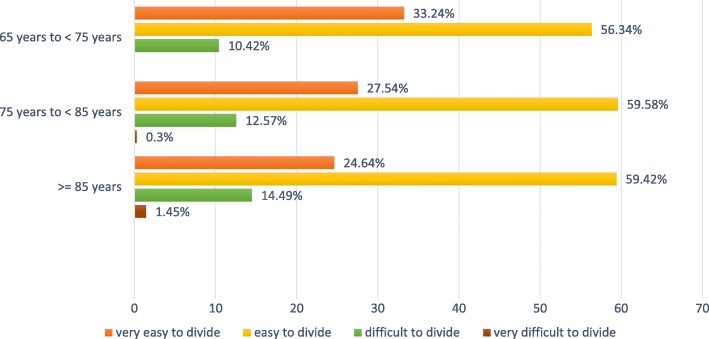
Ease of subdivision analysis (*n* = 758) – Efficacy population. Frequency distribution of the “ease of subdivision rating at Visit 3 or, as applicable, at the time of premature discontinuation of treatment” by age classes

The distribution of this variable is shown by age classes in Fig. 5. Since only 12.01% (91/758) of the patients rated the ease of subdivision as “difficult to divide” or “very difficult to divide”, we constructed a new variable for the analysis by combining these two categories into one category and the other two categories (“very easy to divide” and “easy to divide”) into a second. 87.99% (667/758; 95% CI [85.47%; 90.22%]) of the study participants (≥ 65 years of age) rated the ease of subdivision of the SNAP-TAB tablet into three equal parts as “very easy or easy to divide”. An influence of age or gender of patients on the relative frequency at which the ease of subdivision was rated like this could not be observed (p_Likelihood ratio_ = 0.127).

### Therapy withdrawals and adverse events

Treatment with TC was prematurely terminated in 44 (4.37%) out of 1007 patients recruited. The most common reason for withdrawal was “adverse event” (43.18%) followed by “lack of efficacy” (22.73%).

Overall, the attending physicians documented a total of 110 adverse events in 75 (7.45%) patients of the safety population; 82.72% of these were well-known side effects of TC listed in the SmPC. The most frequently reported AEs were dry mouth (4.87%) and constipation (1.49%). All other documented AEs occurred at a frequency of ≤0.5%. One serious AE was reported (postrenal failure), of which the causality to the study drug was assessed by the physician as unlikely. An at least possible causal relationship between an AE and TC treatment was considered in 97 cases (88.18%) by the investigator, and in 91 cases (82.27%) by the marketing authorization holder. Treatment with the study drug was discontinued in 32 (29.09%) patients, the dosage was reduced in 16 (14.55%) patients, and no measures were taken to treat the AE in 49 (44.55%) cases.

## Discussion

This non-interventional study examined the importance of real-life anticholinergic treatment of elderly outpatients with idiopathic OAB in view of their anticholinergic load and comorbidities, which to our knowledge was not done in any trial before it. The median age of the participants was 75 years; therefore, this population represents the group of adults most frequently affected by the OAB syndrome [13, 27, 48, 49]. 47.54% (445/936) of the elderly in our study were exposed to at least one anticholinergic medication at baseline, as indicated by an ACB scale score of > 0. Thereof 24.72% had an ACB score of ≥3, which is considered by Boustani et al. [19] as clinically relevant. Relating this to the total number of patients in the epidemiological group for which valuable information was available, the percentage of patients with an ACB scale score ≥ 3 was 11.75% (110/936). In a retrospective study of anticholinergic burden in a large cohort of hospitalized geriatric patients (89,579 analysed individuals with a median age of 82 years), 41,456 (46.3%) patients took at least one prescribed anticholinergic drug [50]. Of them, 24,569 (59.3%) had an ACB total score of 1, 5765 (13.9%) a score of 2, and 11,122 (26.8%) a score of 3 or more. Although we observed a population of outpatients in urological practice which is not directly comparable with the hospitalized population in the cohort study, and there is a difference in the median age of the patients in both studies, the magnitude of anticholinergic burden is broadly in line. The observed rate of prescriptions for drugs with anticholinergic properties in our study is also comparable with data from other previous studies, wherein rates vary between 25% in community-dwelling patients to up to 80% in nursing-home residents with cognitive impairments [14, 43, 51].

Several ranked lists have been compiled to assess anticholinergic burden of drugs. The ACB scale identifies the severity of anticholinergic AEs on cognition for many prescribed and over-the-counter medications in a single list [19]. Drugs are rated regarding their anticholinergic burden either through serum anticholinergicactivity or in vitro affinity to muscarinic receptors, and then scored according to their clinical relevance [36]. The study by Pasina et al. [21] found that the ACB scale might help to rapidly identify drugs potentially associated with cognitive impairment in a dose-response pattern.

The epidemiological survey in our study additionally showed a median CIRS-G score-based CMI for all patients of 5 at baseline, and a CMI of > 4 in 52.03% of all patients. The occurrence of a higher comorbidity index, which was found to increase with increasing age, was identified as a potential effect modifier for the ACB scale score, as was age of ≥85 years. These results are supported by an analysis of a large representative primary care dataset (1,751,841 patients) which showed that the prevalence of multimorbidity, defined in this study as the concomitant presence of ≥2 illnesses, increased substantially with age and was present in most people aged 65 years or older [52]. The percentage of subjects with multimorbidity was 64.9% (95% CI [64.7%, 65.1%]) in the group of 65–84 years olds compared to 81.5% (95% CI [81.1%, 81.9%]) in the group of ≥85 years olds. Laux et al. [53] observed a strong correlation between age, gender, multimorbidity (co-occurrence of ≥2 chronic diseases) and health care utilization in the context of the German CONTENT project, analysing the data from 39,699 patients. They discovered that the number of patients’ chronic conditions have a significant impact on the number of different prescriptions (ß = 0.226, *P* < 0.0001) as well as on the number of referrals (ß = 0.3, *P* < 0.0001). In the 60–69 years age group, the average number (± SE) of different prescriptions per patient was 4.45 ± 0.19 in men and 4.55 ± 0.16 in female, whereas the average number of prescriptions rose to 6.51 ± 0.69 and 6.57 ± 0.24, respectively, in the age group ≥80 years.

Currently, there are no CIRS-G based cut-offs for the illness severity and CMI established [54], making the classification of the observed results difficult. Miller et al. [55] identified a mean (± SD) CMI of 4.5 ± 2.5 for a healthy elderly individual with a mean age of 71.1 ± 5.3 years (*n* = 35). In contrast, the total CIRS-G score (± SD) was 12.7 ± 4.7 (*n* = 76) for people with Parkinson’s disease [56]. A CIRS score of ≤6 is used to differentiate between patients who are eligible for intensive chemoimmunotherapy and those who are not [57]. If we apply the cut-off from Miller et al. [55] to our results, then slightly less than half (47.97%) of the study participants in the epidemiological group (473/986) had a CMI score of ≤4, corresponding with that of a healthy elderly subject.

Since anticholinergic activity affects both central and peripheral systems, these drugs are indicated in a wide spectrum of conditions. Multimorbidity and polypharmacy increase the cumulative anticholinergic burden and thus the risk for side effects, including the well-known risk for neurodegenerative disorders [12, 13, 19, 20, 35, 58, 59]. Apart from age-associated changes in pharmacokinetic and pharmacodynamic properties, elderly people may also be more sensitive to anticholinergic effects in the central nervous system because of age-related physiological and pathological changes at the brain [20, 34, 35]. An American population-based study in 12,423 men and women even showed that, comparable to the percentage of anticholinergic users in our study, 47% of the elders (≥ 65 years) used a medication with possible anticholinergic properties, which increased the cumulative risk of cognitive decline and mortality over 2 years in participants with normal or mildly impaired cognition at baseline [18]. Risacher et al. [60] observed that cognitively normal older adults (*n* = 402) taking medications with medium or high anticholinergic activity showed poorer memory and executive function, reduced cerebral glucose metabolism, whole-brain and temporal lobe atrophy, and increased clinical decline compared with non-users; these symptoms were most severe in those with the highest total anticholinergic burden scores. Using data from a well-established prospective cohort study, Chuang et al. [61] discovered that exposure to medications with mild anticholinergic activity in midlife is associated with greater risk of Alzheimer’s disease and accelerated brain atrophy before cognitive impairment. In a population-based, longitudinal study of individuals 65 years or older, higher cumulative use of anticholinergics was associated with an increased risk for all-cause dementia and Alzheimer’s disease [14]. In the cohort study by Pfistermeister et al. [50], anticholinergic drugs with an ACB score of 3 clearly contributed most to the patients’ overall anticholinergic load for all patients having ACB total scores of ≥3. They further found a high anticholinergic burden to be associated with patients with severe cognitive impairment.

In contrast to all tertiary amines indicated in OAB, brain penetration of TC is highly restricted by the molecule’s polar structure and its low lipophilicity, as well as by a P-glycoprotein mediated efflux in the endothelial cells of the blood-brain-barrier (BBB) [62, 63]; in a mouse model, TC permeation across the BBB was not increased with ageing [64]. A recent randomized placebo-controlled clinical study in 59 women aged 50 years and older being treated for OAB with 60 mg of TC per day for 4 weeks, measured no changes in cognitive function between the TC group and the placebo group [65]. Previous clinical studies investigating different indicators for cognitive function and neuropsychological effects, including electroencephalogram and sleep studies, have proven that TC is largely free of CNS effects [66–74]. The drug may therefore provide an effective approach to treating OAB without increasing the patient’s central nervous anticholinergic burden.

This is also supported by the nature and the low frequency of AEs in our study. All documented AEs with an at least causal relationship are well-known side effects of oral TC. In general, TC was well tolerated which is reflected by the subjective assessments of physicians and patients. An observational study of TC 30 mg film-coated tablets in 4092 patients with OAB symptoms achieved comparable results: its tolerability was rated as “very good” or “good” by physicians in 90.2% of cases, and by patients in 87.1% [9].

The relevant changes in characteristic OAB symptoms, observed in the present trial after a median treatment period of 64 days and determined by before-after-comparisons of different variables, indicated that an oral dose of 45 mg TC per day is a potent treatment strategy, providing patients pronounced improvement or cure of the most bothersome symptoms of OAB. This has previously been proven in a previous 12-week, randomised, double-blind, phase IIIb study in 1658 patients with urinary urge incontinence evaluating the efficacy and tolerability of TC which demonstrated that urinary frequency and urge incontinence can be reduced significantly through a flexible dosing strategy [1, 7]. It has further been shown that these clinical effects are associated with improvements in several areas of health-related quality of life of those patients, suggesting a real clinically and personally relevant treatment success [1].

The majority of the patients in our trial (78.55%) - as in the preceding clinical study – were using the approved dose of 45 mg (3 × 15 mg) TC daily, which is defined in the current SmPC [75]. The flexible dosing strategy in our study allowed the physician together with the patient to decide at Visit 2 whether to increase, decrease or maintain the starting dose, or switch to another dosage regimen, until the end of the treatment period. 81.61% (630/772) of patients did not change their initial prescribed daily dose, and only 8.94% (69/772) of patients switched to another dosage regimen. This confirmed that a daily dose of 45 mg of TC is adequate for most patients with idiopathic OAB. This conclusion is supported further by the favourable assessment of efficacy by both the urologists and the patients, as well as the fact that the physicians answered the question relating to therapy continuation affirmatively for 672 out of 752 patients (89.36%) after the observational period. Pooled data from three non-interventional studies in a total of 9366 patients showed that flexible dosing of TC is commonly used in urological practice in Germany [76].

The post hoc analysis in our study, regarding the two dosage schemes “1 x 45 mg TC/day” and “3 x 15 mg TC/day”, did not indicate a difference in clinical outcome for any of the variables. As was further shown in the present study, the SNAP-TAB tablet can be easily divided in three equal units, thereby providing a patient-friendly option in flexible drug dosing. This way of administration simplifies the optimal treatment with TC which should be individualised considering the patient’s comorbidities and comedications, especially in the elderly, and based on the patient’s individual responses to treatment, to ensure an optimal balance between efficacy and tolerability [1, 7–10]. Thus, in turn, it supports close patient adherence and persistence to treatment [76].

Due to its non-interventional and observational character the current trial has distinct advantages and disadvantages associated with it. Limitations are the heterogeneity of participants, the variable dosage regimen and the lack of a comparator group, such as tertiary amines. It must be noted that the epidemiological population in our NIS consisted only of elderly patients (although they account for most patients). While the urologists that attended asked follow-up questions (queries), the possibility that the ACB score data reported by the physicians may have been partly incomplete cannot be ruled out. Missing data for the efficacy analysis was handled by replacing missing values with the corresponding values documented at the last evaluable visit (Last-Observation-Carried-Forward method). Only those patients with evaluable data for the first and last visit and minimum of ≥10 days between the two visits were included in the sensitivity analysis for the variable of interest. This decreased the sample size by approximately 3%. Furthermore, the analysis of the data collected in this NIS and the interpretation of the results could solely be carried out in a descriptive manner.

On the plus side, our study follows national and international recommendations dealing with quality aspects of NIS. With its safety population of 1007 elderly patients and 162 attended urologists nationwide, it comprises a cross-section of the typical population of patients treated for symptoms of idiopathic OAB in daily practice. The open observational scenario highly reflects common use of the study drug. Moreover, the validated instruments, the ACB score and the German version of the CIRS-G scale, used in this NIS cover relevant epidemiological aspects related to the representative patient population. The NIS can therefore be a scientific instrument that completes the results of randomized controlled studies by contributing important data on the use of the drug in real-life practice, for example on medical prescription, dosage recommendations, patients’ compliance, and on safety aspects [77]. The evaluation and demonstration of the adequate patient acceptability of a medicinal product is also presented as a major issue in the EMA Reflection Paper on the Pharmaceutical Development of Medicines for Use in the Older Population [10].

## Conclusions

This NIS focussed on less-known epidemiological issues relating to comorbid conditions and anticholinergic burden from concomitant medications when treating idiopathic OAB. It adds evidence from daily therapeutically practice supporting the favourable benefit-risk profile of TC as reported from the randomized controlled trials. The use of the SNAP-TAB tablet containing 45 mg of TC was shown to be an effective, safe and easy to manage new type of drug administration that facilitates flexible dosing of TC to achieve the optimal patient-related balance between efficacy and tolerability in real-life practice.

## Abbreviations

ACB: Anticholinergic burden
AE(s): Adverse event(s)
AWMF: Arbeitsgemeinschaft der Wissenschaftlichen Medizinischen Fachgesellschaften (Association of the Scientific Medical)
BBB: Blood-brain-barrier
BfArM: Federal Institute for Drugs and Medical Devices
CI: Confidence interval
CIRS-G: Cumulative Illness Rating Scale for Geriatrics
CMI: Comorbidity index
CNS: Central nervous system
DRKS: German Register of Clinical Studies
EMA: European Medicines Agency
MedDRA: Medical Dictionary for Regulatory Activities
NIS: Non-interventional study
OAB: Overactive bladder
OR: Odds ratio
Q1/Q3: 1. quartile / 3. quartile
RSM: Relevant somatic morbidity
SmPc: Summary of Product characteristics
TC: Trospium chloride

## Footnotes

1 Trademark: *Spasmex*® 45 mg film-coated immediate-release tablets (Dr. R. Pfleger GmbH, Bamberg, Germany)
